# Shrimp Shapes a Nitrite Tolerance Trait via Regulating Autophagy and Apoptosis

**DOI:** 10.3390/ijms26041641

**Published:** 2025-02-14

**Authors:** Liping Zhou, Zhentao Ma, Qingyun Liu, Qiangyong Li, Min Peng, Chunling Yang, Bin Zhang, Tiancong Chen, Yuliu Huang, Zhihong Zheng, Aiguo Huang, Xiuli Chen, Yueling Zhang, Xianliang Zhao, Yongzhen Zhao

**Affiliations:** 1 Guangdong Provincial Key Laboratory of Marine Biotechnology, Institute of Marine Sciences, Shantou University, Shantou 515063, China; 20lpzhou@stu.edu.cn (L.Z.); zhengzh@stu.edu.cn (Z.Z.); zhangyl@stu.edu.cn (Y.Z.); 2 School of Marine Sciences, Guangxi Laboratory on the Study of Coral Reefs in the South China Sea, Guangxi University, Nanning 530004, China; 15942391706@163.com (Z.M.); aiguo_200891@126.com (A.H.); 3 Guangxi Academy of Fishery Sciences, Guangxi Key Laboratory of Aquatic Genetic Breeding and Healthy Aquaculture, Nanning 530021, China; 16zyliu2@stu.edu.cn (Q.L.); lqy_cz@163.com (Q.L.); gxnnpm@126.com (M.P.); scsycl@163.com (C.Y.); zhangb41508@163.com (B.Z.); 13977088183@163.com (T.C.); 15878757632@163.com (Y.H.); chenxiuli2001@163.com (X.C.)

**Keywords:** nitrite, *Penaeus vannamei*, tolerance, autophagy, apoptosis

## Abstract

Nitrite is a significant toxic substance that causes acute toxicity in aquatic animals. However, the molecular mechanisms underlying nitrite tolerance remain largely unidentified. Here, we investigated the physiological and transcriptomic profiles of nitrite-sensitive and nitrite-tolerant families of the white shrimp *Penaeus vannamei* under nitrite stress. Exposure to a lethal concentration of nitrite for 72 h caused higher mortality, higher nitrite accumulation, and more severe physiological damage in both the hemolymph and hepatopancreas of nitrite-sensitive families than in nitrite-tolerant families. This damage manifested as hepatic tubular deformation, hepatocyte necrosis, decreased hemocyte counts, lowered phagocytic activity in hemocytes, elevated production of reactive oxygen species (ROS), and decreased AKP enzyme activity. Furthermore, transcriptomic analysis revealed an upregulation in protein processing in the endoplasmic reticulum (ER) in the nitrite-tolerant families, triggering apoptosis- and autophagy-related pathways in the hemocytes and hepatopancreas upon nitrite exposure. Additionally, TUNEL staining and transmission electron microscopy (TEM) experiments revealed that the tolerant families may mitigate nitrite toxicity by modulating autophagy and apoptosis. Collectively, our results provide a valuable foundation for exploring the molecular mechanisms underlying nitrite tolerance in shrimp, which could facilitate the targeted breeding of shrimp germplasm with enhanced nitrite tolerance in aquaculture.

## 1. Introduction

The white shrimp *Penaeus vannamei* farming industry has experienced rapid expansion due to its adaptation to a broad range of salinity and considerable benefits [1,2,3]. Nonetheless, the aquaculture industry’s development is impeded by the increased nitrite concentrations associated with the widespread adoption of high-density farming practices [2]. In the later stages of cultivation, this issue can become more prominent. The main cause of this problem arises from the breakdown of remaining feed by nitrifying bacteria [3,4,5]. Simultaneously, as plankton populations increase, the amount of dissolved oxygen in the aquatic environment decreases, affecting the growth and activity of nitrifying bacteria and causing a disruption in the conversion of nitrite [6,7]. Additionally, shrimp excretion contains a high amount of organic nitrogen that can be transformed into nitrite by nitrifying bacteria, thereby presenting a high level of nitrite [8]. Research on marine organisms have demonstrated that nitrite concentrations in the Xuwei River in Lianyungang City of China ranged from 0.15 to 1.02 mg/L [8]. In intensive aquaculture or recirculated water systems, nitrite concentrations can exceed 50 mg/L [9], significantly exceeding the real safety level of 2.55 mg/L [10]. Nitrite is able to enter aquatic animals through their gills and intestines [11,12]. Upon entering the body, nitrite can disrupt the valence state of copper ions in hemocyanin through oxidation [13], impairing its oxygen-binding capacity and resulting in tissue hypoxia in shrimp [14]. Aquatic animals are highly vulnerable to the toxic effects of nitrite, such as nitrogen excretion [15], endocrine disruption [16], ionic balance [17], and oxygen transport [18]. The effects of nitrite on aquatic animals vary significantly based on their sensitivity and physiological responses. Crustaceans, such as *Penaeus vannamei*, have a high sensitivity to nitrite, with a safety threshold as low as 0.53 mg/L, due to their exposed gills [19] and increased oxygen demands [20]. Similarly, *Eriocheir sinensis* show have a high sensitivity to nitrite with a safety threshold as low as 2.6 mg/L. The nitrite stress can disrupt the structure of gill tissues, causing osmoregulation disturbances and immune dysfunction [21]. However, fish species have acclimatization mechanisms with nitrite exposure. Kim et al. reported that juvenile hybrid groupers (*Epinephelus lanceolatus* ♂ × *Epinephelus fuscoguttatus* ♀, Family: Serranidae) exhibit a high tolerance to nitrite stress up to 20 mg/L, due to their robust detoxification mechanisms [22]. Up to now, the disruptions of nitrite have been extensively investigated in various studies, such as in a model of nitrite-stressed grass crabs (*Ctenopharyngodon idella*), which was found to induce apoptosis by activating the IRE1 signaling pathway associated with ER stress [23]. Moreover, nitrite exposure also caused oxidative stress in blue crabs, resulting in the suppression of antioxidant enzyme activity, which in turn promoted apoptosis and DNA damage [24]. Interestingly, certain shrimp species have exhibited a remarkable capacity to endure nitrite stress, despite the significant challenges it poses. However, further research is still needed regarding the mechanisms that enable shrimp to withstand nitrite stress.

Tolerance to nitrite substances is acknowledged to be a complex mechanism. Several efforts have been launched to shed light on the physiological toxicity and immune response in aquatic species subjected to nitrite stress [25,26]. Prior studies have demonstrated that nitrite exposure could induce oxidative stress, DNA damage, and apoptosis in mud crab (*Scylla paramamosain*) [24] and dysfunction and oxidative damage in grass carp (*Ctenopharyngodon idella*) [27]. In shrimp, we have preliminarily proved that the nitrite tolerance of shrimp is related to their antioxidant ability [28], indicating that improving antioxidant capacity can alleviate the oxidative stress caused by nitrite. Information indicated that reactive oxygen species (ROS) was generally accompanied by oxidative stress [29]. To maintain the homeostasis, shrimp eliminate ROS to prevent or repair oxidant damage by their strong antioxidant defense system, including antioxidant enzymes, such as superoxide dismutase (SOD) [30], as well as their nonspecific immune system, such as acidic phosphatase (ACP) and alkaline phosphatase (AKP) [31]. Although it is known that major toxicity of nitrite and host’s immune response of shrimp under nitrite stress is altered. However, there is still a significant lack of studies on the physiological and differential transcriptomic responses of multiple tissues to elucidate the mechanisms that enable shrimp to endure such stress.

For crustaceans, the hepatopancreas is particularly vulnerable to the effects of toxins due to its role as the primary organ for metabolism and detoxification, dealing with heavy metals, harmful compounds, and pesticides [32,33]. In addition, shrimp hemolymph is crucial for detoxification and immune defense, functioning as sites for nitrite accumulation during exposure to these toxic agents [15]. Nevertheless, the mechanisms related to immune tolerance of hemocytes and the hepatopancreas in shrimp under nitrite stress remain unclear. In our previous research, we observed that high expression levels of immune and ER-related genes may serve as an important adaptive strategy for nitrite-tolerant families in comparison to nitrite-sensitive families [34]. In order to confirm this finding, more investigation is necessary to understand the physiological and immune response in hemocytes and the hepatopancreas to nitrite stress. Therefore, this study employed nitrite-tolerant and nitrite-sensitive families as a model to facilitate a comprehensive analysis of toxicity and tolerance mechanisms during nitrite stress. This can help understand the molecule changes in response to nitrite resistance. Furthermore, a comparative analysis of hemocytes and hepatopancreas transcriptomes can offer valuable information on evolutionary adaptations and shared mechanisms that help organisms cope with nitrite stress. Ultimately, we speculated that protein processing in the endoplasmic reticulum, autophagy, and apoptosis were identified as crucial factors in nitrite tolerance. These findings will enhance our understanding of the molecular basis for nitrite susceptibility or tolerance in *Penaeus vannamei* and facilitate the implementation of more refined breeding techniques for the aquatic industry.

## 2. Results

### 2.1. Physiological Changes of Nitrite-Tolerant and Nitrite-Sensitive Families Under Nitrite Stress

**Figure 1:**
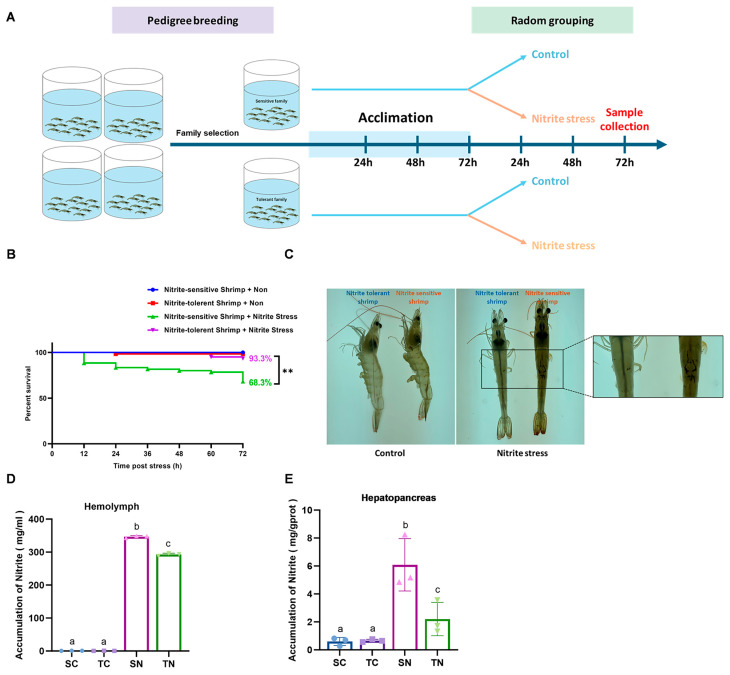
Effects of nitrite stress on sensitive and tolerant families. (**A**) Design and treatment of groups for experiment (SC: nitrite−sensitive families control subgroup, TC: nitrite−tolerant families control subgroup, SN: nitrite−sensitive families stress subgroup, and TN: nitrite−tolerant families stress subgroup). (**B**) The survival rate of shrimp after nitrite stress (the survival data were analyzed by the log−rank test and visualized with a *Kaplan-Meier* plot), “**” for *p* < 0.01. (**C**) The appearance of shrimp after nitrite stress. Accumulation of nitrite in (**D**) hemolymph and (**E**) hepatopancreas. The results from three independent replicates were analyzed by one−way ANOVA and two−way ANOVA (Tukey’s test, *p* < 0.05). There were significant differences in the representation of different letters on the histogram (*p* < 0.05).

After 10 generations of selective breeding, two distinct families of *Penaeus vannamei* have been established, displaying either tolerance or sensitivity to nitrite. The median lethal concentrations (LC_50_) of both tolerant and sensitive families were assessed over a 96 h period. The results showed that the tolerant families exhibited significantly higher LC_50_ values at 24, 48, 72, and 96 h compared to the sensitive families. Specifically, the LC_50_ of nitrite in the tolerant families was 747 mg/L, markedly higher than the 359 mg/L observed in the sensitive families over 96 h (Figure S1). Following a three-day acclimation period, individuals from both families were randomly divided into two subgroups: one subgroup experienced immersion stress with nitrite, while the other served as a control under standard culture conditions for an additional three days (Figure 1A). As expected, the nitrite-tolerant families demonstrated a notable survival rate of 93.3%, significantly higher than the 68.3% survival rate in the sensitive families (*p* < 0.05, *n* = 60) (Figure 1B). Moreover, the nitrite-sensitive families exhibited pronounced physiological damage upon nitrite exposure, including severe shell injuries, blackening, and muscle cloudiness (Figure 1C). Both the TN and SN groups exhibited notably elevated nitrite concentrations in their hemolymph and hepatopancreas under nitrite stress, with the TN group displaying comparatively lower accumulation (*p* < 0.05) (Figure 1D,E). These findings under the ability of the nitrite-tolerant families to withstand nitrite stress, providing them significant advantages over sensitive families.

**Figure 2:**
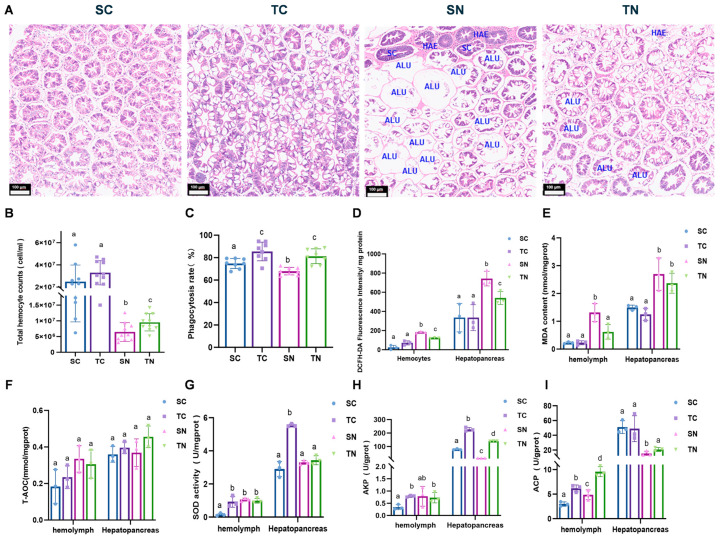
Oxidative damage parameter changes of *Penaeus vannamei* under nitrite stress. (**A**) Histology of hepatopancreas of shrimp (200×). The letters in the figure indicated that: SC: swelling cells; ALU: abnormal lumen; HAE: hemolymphatic embed; scale bar = 100 μm. (**B**) Effects of nitrite stress on total hemocyte counts (THCs). (**C**) Effects of nitrite stress on phagocytosis rate of hemocyte. (**D**) Effects of nitrite stress on ROS production. (**E**) Effects of nitrite stress on Malondialdehyde (MDA) content. (**F**) Effects of nitrite stress on the total antioxidant capacity (T-AOC). (**G**) Effects of nitrite stress on superoxide dismutase activity (SOD). (**H**) Effects of nitrite stress on alkaline protease activity (AKP). (**I**) Effects of nitrite stress on acid Phosphatase activity (ACP). SC: nitrite−sensitive families control subgroup; TC: nitrite−tolerant families control subgroup; SN: nitrite−sensitive families stress subgroup; TN: nitrite−tolerant families stress subgroup. There were significant differences in the representation of different letters on the histogram (*p* < 0.05). The results from three independent replicates were analyzed by one−way ANOVA (Tukey’s test, *p* < 0.05) and shown as the mean + SD (*n* = 3).

The impact of nitrite stress on the structural integrity of shrimp hepatopancreas was assessed by examining histological features using H&E staining after nitrite stress of 72 h. The hepatopancreas tissue under nitrite stress displayed significant damage compared to the control group, characterized by the dilated renal tubule lumens, epithelial vacuolization, hepatocyte swelling, and hemocyte infiltration. Particularly, increased hepatocyte necrosis was observed in the nitrite-sensitive group. However, the nitrite-tolerant families exhibited significantly reduced tissue damage compared to the nitrite-sensitive families (Figure 2A). Following a 72 h nitrite stress, both families showed a notable decrease in hemocyte quantity and phagocytic function. Nonetheless, the tolerant group demonstrated higher hemocyte counts and enhanced phagocytic capabilities compared to the nitrite-sensitive families under nitrite stress (*p* < 0.05) (Figure 2B,C). Additionally, elevated levels of reactive oxygen species (ROS) and malondialdehyde (MDA) were observed in both hemocytes and hepatopancreas following nitrite exposure, with the tolerant group displaying significantly lower levels of ROS and MDA compared to the sensitive group (*p* < 0.05) (Figure 2D,E). No significant difference (*p* > 0.05) was observed in A-TOC levels in the hemolymph and hepatopancreas between the treated and control groups following a 72 h nitrite stress (Figure 2F). In the absence of nitrite stress, the SOD enzyme activity in the hemolymph and hepatopancreas of tolerant families was significantly higher than that of the sensitive families (*p* < 0.05). However, under nitrite stress, there was no significant differences between the two families (Figure 2G). Further analysis revealed that the alkaline phosphatase (AKP) activity in the hemolymph and hepatopancreas of the nitrite-resistant group was significantly higher than that in the nitrite-sensitive group (*p* < 0.05). During nitrite stress, plasma AKP activity increased, while the AKP enzyme activity in the hepatopancreas was suppressed in the nitrite-sensitive group (*p* < 0.05) (Figure 2H). Furthermore, the resistant families demonstrated elevated acid phosphatase (ACP) enzyme activity in their hemolymph compared to the sensitive families, both under normal conditions and nitrite-induced stress (*p* < 0.05). Conversely, the ACP enzyme activity in the hepatopancreas significantly decreased following exposure to nitrite stress (*p* < 0.05) (Figure 2I). These findings indicated that nitrite stress can increase oxidative damage and impair immune enzyme functions in the shrimp hemolymph and hepatopancreas, and the nitrite-tolerant families exhibit less stress toxicity compared to the nitrite-sensitive families.

### 2.2. Differential Transcriptomic Profiles in Nitrite-Tolerant and Sensitive Families

Transcriptome analyses were conducted on the hemocytes and hepatopancreas of shrimp from nitrite-sensitive and nitrite-tolerant families following nitrite exposure to investigate the mechanisms underlying nitrite tolerance. The sequencing results revealed a total of 48,020,684 and 47,659,456 clean reads (exceeding 95%) in the hemocytes and hepatopancreas, respectively. Each sample generated clean data exceeding 6.01 Gb, with the percentage of Q30 bases surpassing 95.14%. Subsequently, the clean reads of each sample were aligned with the designated reference genome individually, resulting in alignment rates ranging from 81.57% to 89.18% (Table S2). The raw data have been deposited in the National Center for Biotechnology Information (NCBI) Sequence Read Archive (SRA) under the BioProject number PRJNA1154976.

**Figure 3:**
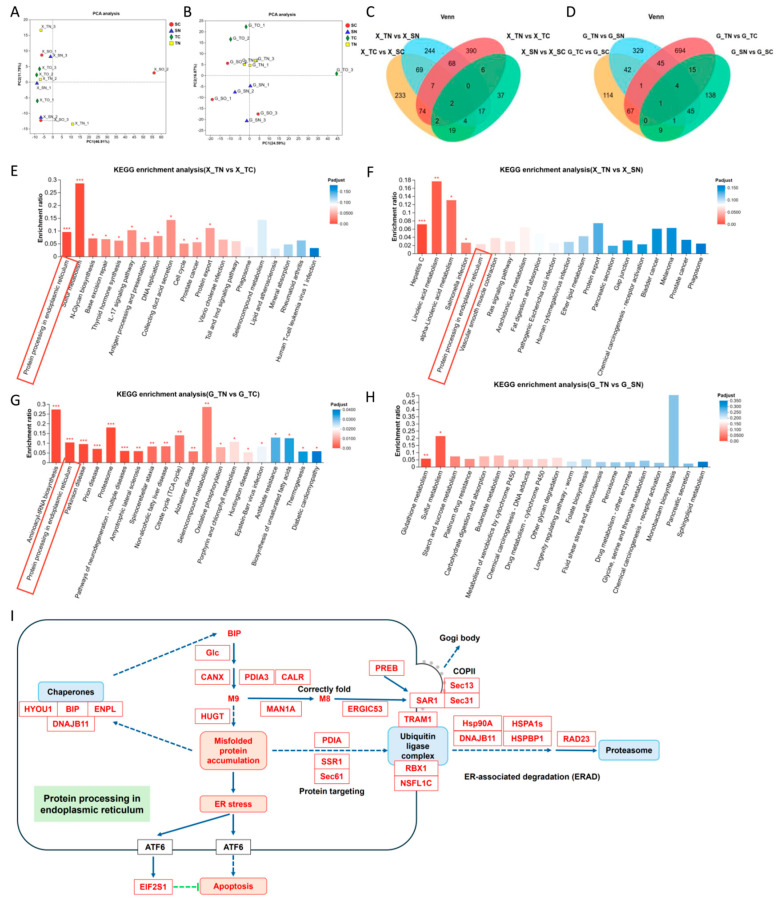
Different KEGG−enriched genes between nitrite-sensitive families and nitrite−tolerant families in hemocytes and hepatopancreas under nitrite stress for 72 h. PCA score diagram of transcriptomic data in (**A**) hemocytes and (**B**) hepatopancreas. Each point represents a specific sample. The Venn diagram of DEGs in (**C**) hemocytes and (**D**) hepatopancreas. KEGG analysis of upregulated DEGs of hemocytes in (**E**) TN vs. TC comparison and (**F**) TN vs. SN comparison. KEGG analysis of upregulated DEGs of hepatopancreas in (**G**) TN vs. TC comparison and (**H**) TN vs. SN comparison. (**I**) DEGs enriched to protein processing in endoplasmic reticulum in hemocytes and hepatopancreas. Red characters denote upregulated genes, while black characters indicate genes with insignificant changes. A solid arrow represents a direct effect, whereas a dashed arrow signifies an indirect effect. SC: nitrite−sensitive families control subgroup, TC: nitrite−tolerant families control subgroup, SN: nitrite−sensitive families stress subgroup, and TN: nitrite−tolerant families stress subgroup. Significant differences between groups were denoted with “*” for *p* < 0.05, “**” for *p* < 0.01 and “***” for *p* < 0.001.

Principal component analysis (PCA) revealed distinct sample clustering among different families under various conditions. Among hemocytes and the hepatopancreas, the sensitive families exhibited distinct differences from tolerant families (Figure 3A,B). However, notable differences were noted among the four groups in the hepatopancreas, whereas the four groups in hemocytes exhibited relatively close similarity. These results highlight both commonalities and differences in the transcriptome data. A Venn diagram concisely displays the variations in gene expression between stress and control groups within individual families. Following nitrite stress, a comparative analysis between sensitive and tolerant families identified 78 commonly differentially expressed genes in hemocytes and 44 in the hepatopancreas. In addition, 10 common differentially expressed genes were found in comparisons between the TN and TC groups, and 20 were identified in the SN and SC groups, respectively (Figure 3C,D). Notably, most differentially expressed genes were specific to each comparison group, indicating considerable differences in gene expression profiles between the two families. To verify the reliability of the transcriptome sequencing data, eight DEGs associated with autophagy and apoptosis were chosen from the transcriptome database for RT-qPCR analysis. The results demonstrated consistent expression changes between the transcriptome data and the RT-qPCR data for all genes (Figure S2), affirming the reliability of the transcriptome’s differential expression analysis. Based on these results, we speculated that the sensitive families exhibit distinct differences from tolerant families during nitrite stress.

### 2.3. Protein Processing in the Endoplasmic Reticulum Is Overexpressed in Tolerant Families Under Nitrite Stress

To investigate pathways associated with nitrite tolerance, an analysis of KEGG functional enrichment analysis was carried out, with particular emphasis on genes belonging to the tolerant families that exhibited notable upregulation in response to nitrite stress, as well as genes from the tolerant families displaying enhanced sensitivity in comparison to those exposed to nitrite stress. Within hemocytes, the genes upregulated in the tolerant families were significantly enriched in the pathways of protein processing in the endoplasmic reticulum (map04141) and sulfur metabolism (map00920) (Figure 3E, Table S3). In contrast to the sensitive families, the tolerant families exhibited a significant upregulation of genes associated with hepatitis C (map05160) and linoleic acid metabolism (map00591) (Figure 3F, Table S4). Within the hepatopancreas, the genes that were upregulated in the tolerant families under nitrite stress were notably enriched in aminoacyl-tRNA biosynthesis (map00970) and protein processing in the endoplasmic reticulum (map04141) (Figure 3G, Table S5). In comparison to sensitive families, the tolerant families exhibited a distinct enrichment of genes significantly upregulated in glutathione metabolism (map00480) and sulfur metabolism (map00920) (Figure 3H, Table S6). Notably, an increase in protein processing in the endoplasmic reticulum was significantly observed in both types of tissues in tolerant families, suggesting a potential association with nitrite tolerance. KEGG enrichment analysis was performed on genes significantly upregulated in both hemocytes and hepatopancreas of tolerant families before and after nitrite stress. The results showed that the differentially expressed genes (DEGs) co-upregulated in both tissues were mainly enriched in protein processing within the endoplasmic reticulum (map04141). Targeting misfolded proteins such as *PDIA*, *SSR1*, and *Sec61*, along with endoplasmic reticulum-associated degradation (ERAD) components *HSP90A*, *HSPA1s*, *DNAJB11*, *HSPBP1*, and *RAD23* showed notable upregulation (Figure 3I). Thus, we hypothesized that nitrite-tolerant families may regulate protein processing in the endoplasmic reticulum to shapes a nitrite tolerance trait.

### 2.4. Effect of Nitrite Stress on Apoptosis and Autophagy Between Nitrite-Tolerant and Sensitive Shrimp Families

**Figure 4:**
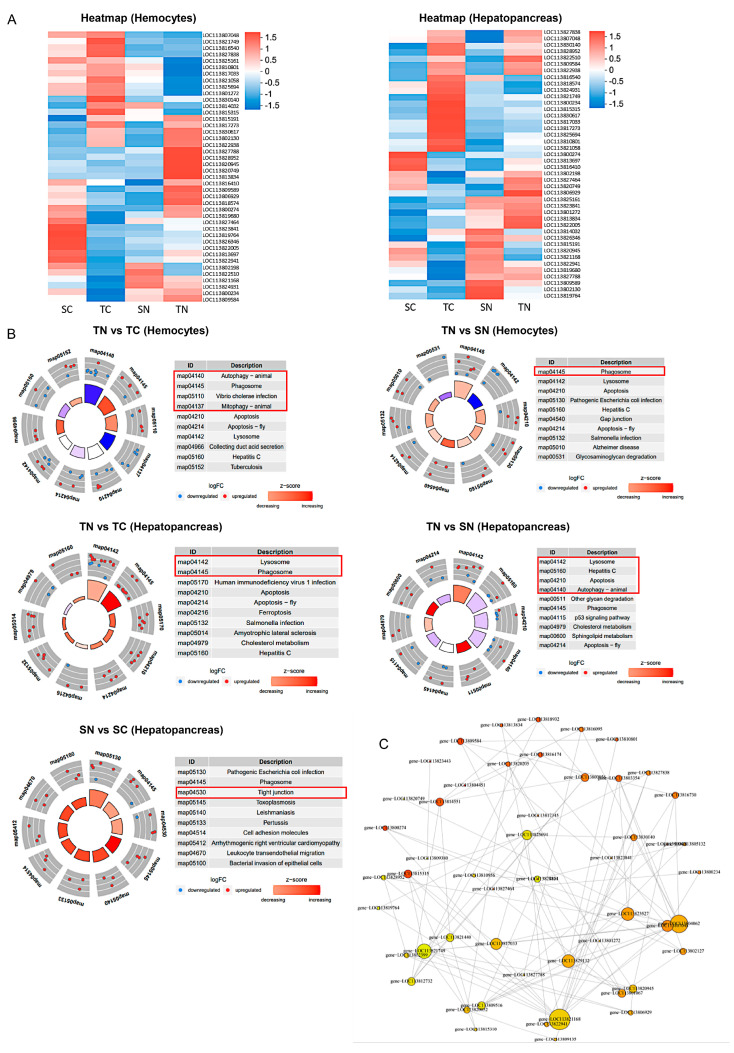
Alteration of apoptosis− and autophagy−related genes in hemocytes and hepatopancreas under nitrite stress. (**A**) Heatmap of transcriptomics. Each column represents a group, and each row represents a transcript. Blue to red colors corresponds to low to high abundance, respectively. (**B**) KEGG pathway enrichment analysis of autophagy− and apoptosis−related genes in hemocytes and hepatopancreas of nitrite−tolerant and nitrite−tolerant families under nitrite stress. Blue dots indicated downregulated DEGs, red dots represented upregulated DEGs. (**C**) Interactive analysis of autophagy− and apoptosis−related genes in hemocytes and hepatopancreas. Different nodes of the diagram display different colors. Size of the dot represents the influence of gene. SC: nitrite−sensitive families control subgroup, TC: nitrite−tolerant families control subgroup, SN: nitrite−sensitive families stress subgroup, and TN: nitrite−tolerant families stress subgroup.

Unfolded proteins may accumulate during protein processing in response to external stimuli, resulting in endoplasmic reticulum (ER) stress, subsequently triggering apoptosis and autophagy [26]. To evaluate the effect of nitrite stress, we compared apoptosis levels between the two families. A clustered heat map was used to depict differentially expressed genes (DEGs) associated with apoptosis and autophagy in hemocytes and the hepatopancreas. Remarkably, a significant enrichment of these genes was observed in tolerant shrimp exposed to nitrite stress (Figure 4A). Following this, a KEGG enrichment analysis was performed on the significantly changed DEGs. The results indicated a significant suppression of the autophagy-animal (map04140) pathway and a notable enhancement of the phagosome (map04145) pathway in hemocytes from tolerant families following exposure to nitrite stress. In the hepatopancreas, it was observed that the expression of the lysosome (map04142) and autophagy animal (map04140) pathways significantly increased in tolerant shrimp families both pre- and post-nitrite stress. In contrast, the hepatopancreas of the sensitive families exhibited a significant upregulation of the lysosome (map04142) pathway in response to nitrite stress. This upregulation further stimulated the expression of genes associated with the tight junction (map04530) pathway both pre- and post-nitrite stress (Figure 4B). Analysis of the interactions among these differentially expressed genes related to autophagy and apoptosis highlighted the significant role of calnexin (LOC113804062), as depicted in Figure 4C. These results indicate that the tolerant families mainly engages in the mechanism of nitrite tolerance through the modulation of autophagy- and apoptosis-related pathways in the hepatopancreas upon nitrite exposure.

### 2.5. Apoptosis and Autophagy Analysis of Tolerant and Sensitive Shrimp Families Under Nitrite Stress

**Figure 5:**
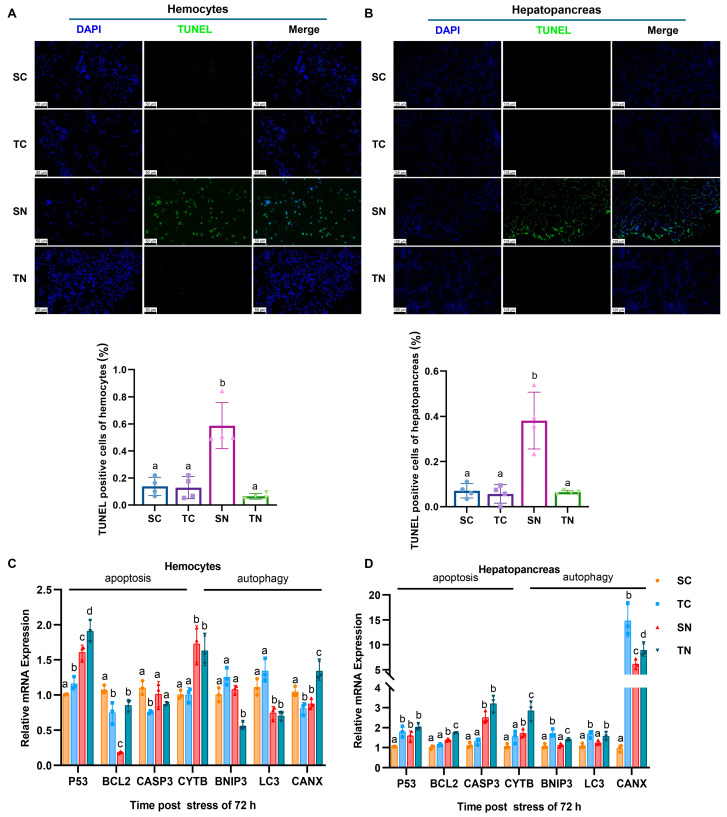
Nitrite stress promoted apoptosis in nitrite−sensitive families and induces autophagy in nitrite-tolerant families. Apoptosis analysis of *Penaeus vannamei* in (**A**) hemocytes and (**B**) hepatopancreas under nitrite stress. Common cell nuclei appeared blue; TUNEL−positive cell nuclei appeared green. Scale bars are 50 μm in hemocytes and 125 μm in hepatopancreas. The expressions of apoptosis- and autophagy-related genes in (**C**) hemocytes and (**D**) hepatopancreas under nitrite stress. There were significant differences in the representation of different letters on the histogram (*p* value < 0.05). SC: nitrite−sensitive families control subgroup, TC: nitrite−tolerant families control subgroup, SN: nitrite−sensitive families stress subgroup, and TN: nitrite−tolerant families stress subgroup. The results from three independent replicates were analyzed by one−way ANOVA (Tukey’s test, *p* < 0.05) and shown as the mean + SD (*n* = 3).

TUNEL staining was utilized to detect apoptotic cells in hemocytes and the hepatopancreas. The results revealed a notably higher level of hemocyte apoptosis in the SN group compared to the TN group under nitrite-induced stress (*p* < 0.05) (Figure 5A). Furthermore, significant apoptosis was observed in the hepatopancreas of the SN group in comparison to the control group (SC) (*p* < 0.05) (Figure 5B). Moreover, an increase in the expression of the apoptosis-related gene P53 and the autophagy gene *CYC* was observed in hemocytes, and a decrease in the expressions of the apoptosis gene *Bcl2* and the autophagy-related gene *LC3* (*p* < 0.05) (Figure 5C). The hepatopancreas exhibited a significant upregulation of apoptosis-associated genes (*P53*, *Bcl2*, *CASP3*), particularly in tolerant families. Furthermore, there was a notable increase in the expression of autophagy-related genes (*CYC*, *BNIP3*, *LC3*) in tolerant families compared to sensitive families (*p* < 0.05) (Figure 5D).

**Figure 6:**
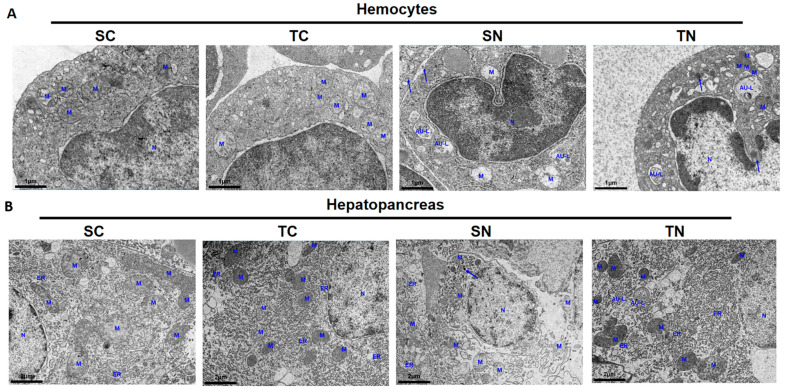
Histopathological analysis of sensitive and tolerant families in hemocytes and hepatopancreas under nitrite stress for 72 h by TEM. (**A**) In hemocytes. (**B**) In hepatopancreas. M, mitochondria; N, nucleus; ER, endoplasmic reticulum; AU-L, autophagy lysosome; blue arrows represent apoptotic body. SC: nitrite−sensitive families control subgroup, TC: nitrite−tolerant families control subgroup, SN: nitrite−sensitive families stress subgroup, and TN: nitrite−tolerant families stress subgroup. The results from three independent replicates.

Autophagy is widely recognized for its essential function in degrading misfolded proteins. Transcriptomic analysis revealed a notable increase in phagosome-associated pathways in the TN group. Subsequently, we employed transmission electron microscopy to examine hemocytes and hepatopancreas responses to nitrite-induced stress (Figure 6A,B). When exposed to nitrite stress, chromatin in the hemocytes and hepatopancreas of shrimp experienced condensation and aggregation, leading to the generation of apoptotic bodies of different sizes enclosed by membranes. Furthermore, there was a notable increase in the number of autophagosomes, characterized by double or multilayer membranes enclosing cytoplasmic components, and autolysosomes with single-layer membranes. Conversely, a significant decrease in mitochondrial quantity was observed, accompanied by structural abnormalities including swelling, shrinkage, and the absence of cristae. Particularly, an elevated incidence of mitochondrial vacuolization was observed in the hemocytes and hepatopancreas of the SN group, whereas the nitrite-tolerant lineage displayed a higher presence of autolysosomes. Generally, the hemocytes and hepatopancreas of the TN group retained mitochondria with a relatively complete structure, along with an abundance of endoplasmic reticulum and ribosomes. Under nitrite stress, it is crucial to maintain intact mitochondria and endoplasmic reticulum to support the energy metabolism and immune homeostasis of nitrite-tolerant species, thereby mitigating the damage induced by this stress. The findings indicate that the tolerant families have higher autophagy in the hepatopancreas and lower apoptosis in hemocytes compared to the sensitive families, triggering the ability of the nitrite-tolerant families to withstand nitrite stress.

## 3. Discussion

The toxicity by nitrite is typically manifested by inhibiting the growth of shrimp [10], causing oxidative stress [25], and ultimately resulting in their death [14]. As nitrite continuously enters the hemolymph through the gills and spreads to the entire body, its toxicity to aquatic animals, particularly shrimp, is gradually intensified [19]. Due to the continuous accumulation of nitrite, ROS are produced, which may cause hemocytes to rupture and swell [26,35,36,37]. Moreover, nitrite stress significantly reduces the phagocytic rate of hemocytes, resulting in immune suppression and metabolic disorders [26,38]. High levels of nitrite can lead to acute toxicity in shrimp, and potentially high mortality rates within a short period [14]. Nitrite stress affects the phagocytic signal transduction of cells by inhibiting the release of intracellular calcium ions [39], which renders shrimp more susceptible to pathogens [14,26,28,40]. Furthermore, nitrite stress can lead to decreased food intake and metabolic disorders in shrimp, further negatively impacting growth and survival rates of shrimp [3]. However, it is still uncertain whether the higher survival rates in the nitrite-tolerant families are due to inherent defense mechanisms or differences in inducible responses after nitrite exposure, as noted in other biological systems [41,42].

In this study, we investigated hemolymph and hepatopancreas differences in selectively shrimp to understand physiological differences between tolerant and sensitive phenotypes. For the nitrite-tolerant families, shrimp exhibited a remarkable 93.3% survival rate after enduring acute nitrite stress for 72 h. Conversely, the nitrite-sensitive families experienced a notable decrease in survival rate to 68.3%, coupled with visible physiological damage including darkening and damage to the shell, blanching of muscle tissue, and yellowing of gill filaments. In addition, nitrite is highly accumulated in hemolymph and hepatopancreas, especially in sensitive families (Figure 1D,E). Consistent with prior research, these findings indicate nitrite accumulation in the hemolymph and hepatopancreas of *Penaeus monodon* [43]. This indicates the achievement of establishing nitrite-tolerant families and a nitrite-sensitive families, laying a valuable foundation for further investigations into shrimp nitrite tolerance. At 96 h, the LC50 values for sensitive and tolerant families of *Penaeus vannamei* were 359 mg/L and 747 mg/L, respectively. These values are comparable to those reported for *Danio rerio* (411 mg/L) [44], *Chitala ornata* (539.6 mg/L) [45], and *Anodonta woodiana* (618.7 mg/L) [46], but much higher than values reported for other aquatic species, such as *Eriocheir sinensis* (38.9 mg/L) [21], *Pangasianodon hypophthalmus* (113.85 mg/L) [47], *Pleoticus muelleri* (157 mg/L) [48], and *Nibea albiflora* (99.08 mg/L) [49].

In many studies of nitrite-stressed aquatic animals, researchers have found that nitrite stress results in the significant dissolution of epithelial cells in hepatic tubules and the presence of obvious vacuoles, which may impair the immune and metabolic functions of the hepatopancreas [25,50,51]. In our study, we noted significant histological damage in the hepatopancreas of these sensitive families exposed to nitrite stress, including cellular swelling, the partial disruption of structures, and the loss and necrosis of lumen stellate structures (Figure 2A), which is a previously discovered toxic feature of nitrite [52]. *Penaeus vannamei*, like other invertebrates, relies on its innate immune response as the primary defense mechanism against environmental stressors [53,54]. In addition to humoral immunity, invertebrates utilize cellular immunity to mount innate immune responses to such stressors. In our research, tolerant families showed a higher count of hemocytes and improved phagocytic activity compared to the sensitive families in healthy conditions, which has been proved to be crucial for cellular immunity in previous studies [55], suggesting that tolerant families can adeptly manage potential stress. Moreover, oxidative stress is a severe imbalance between the generation of ROS and antioxidant defense in favor of ROS, resulting in excessive oxidative damage to biomolecules [56]. Previous studies have indicated that excessive ROS leads to elevated levels of MDA in the organism [57], which is an indicator of the extent of cellular oxidative damage [58]. When the level of ROS in the body is too high, aquatic animals rely on their antioxidant systems to eliminate ROS and MDA and maintain antioxidant homeostasis [59]. It has been reported that SOD is the primary enzyme responsible for removing ROS from cells [60,61], while ACP and AKP function as pivotal lysosomal enzymes for aquatic organisms to measure the immune and health status of shrimp, playing a vital role in non-specific immune defense against environmental stressors [62]. Compared with the sensitive family, the significant decrease in ROS and MDA levels in the tolerant family in this study can be largely attributed to the enhanced activities of SOD, AKP, and ACP, which collectively provide superior protection compared to the sensitive family and aligns with the study of detoxifying nitrite by upregulating SOD activity in Pacific white shrimp [63]. These results indicate that the nitrite-tolerant families display enhanced immune functionality in response to elevated nitrite stress.

Upon encountering external triggers, cells undergo a range of adaptive modifications designed to cope with injuries and enhance tolerance. Conversely, once the damage is deemed irreparable, cells often trigger apoptosis to safeguard the overall cellular system from additional injury [64]. Common stimuli include heat stress, oxidative stress, hypoxic stress, and DNA damage, which rapidly disrupt the protein folding capacity of the ER and induce the accumulation of misfolded and unfolded proteins in the ER [65]. In reaction to stress, cells trigger the unfolded protein response (UPR) to restore ER homeostasis and facilitate protein refolding [66,67]. When these pathways are unable to alleviate ER stress stemming from the accumulation of abnormal proteins, apoptosis and autophagy is triggered [68,69]. In our study, compared to nitrite-sensitive families, we observed that nitrite-tolerant families exhibited a significant upregulation of luminal chaperones (*HYOU1*, *Bip*, *ENPL,* and *DNAJB11*) involved in ER protein processing in their hemocytes and hepatopancreas after nitrite stress (Figure 3), which may help trigger the UPR to preserve cell integrity by activating autophagy [70]. In previous studies, *BIP*, also known as 78 kDa glucose-regulated protein (GRP78), serves as the primary regulator of the UPR in the endoplasmic reticulum. Moreover, key genes for recognizing UPR of shrimp such as *PDIA*, *SSR1,* and *Sec61,* were found to be significantly upregulated under nitrite stress, which were reported to alleviate oxidative stress-induced damage [71,72,73]. Concurrently, genes associated with the degradation of misfolded proteins, including *TRAM1*, *RBX1*, *NSFL1C*, *Hsp90A*, *HSPA1s*, *DNAJB11*, *HSPBP1*, and *RAD23*, exhibited upregulation (Figure 4). All these UPRs are dedicated to preserving ER homeostasis, which have been widely recognized [74]. In previous research, numerous studies have reported that ER stress destroys the immune homeostasis of aquatic animals, such as in *Danio rerio* subjected to MC-LR and nitrite [36,75], in *Ctenopharyngodon idella* under nitrite stress [23], and in *Penaeus vannamei* exposed to ammonia [76]. Therefore, maintaining endoplasmic reticulum homeostasis is essential for shrimp survival under nitrite stress.

Furthermore, we observed an increase in autophagic lysosomes and the appearance of apoptotic vesicles in shrimp hemocytes and hepatopancreas after nitrite stress by TEM, accompanied by the upregulation of genes implicated in apoptosis and autophagy (Figure 6). In shrimp, a complex relationship exists among ROS, autophagy, and apoptosis [77]. Under conditions of oxidative stress, autophagy plays a crucial role in removing damaged mitochondria, reducing ROS production, and inhibiting apoptosis, thereby protecting cells from further damage [78]. Elevated concentrations of ROS can induce apoptosis in hemocytes, leading to a decrease in their numbers and a reduction in the phagocytosis rate [79], which is consistent with our research. Under nitrite stress, mitochondria suffered significant damage, and the level of ROS in nitrite-sensitive families increased, and then the number of hemocytes decreased, along with a reduction in their phagocytosis rate. Conversely, moderate levels of autophagy can help maintain the healthy state of nitrite-tolerant families, thereby sustaining a high phagocytosis rate and enhancing the immune response. Moreover, the significant apoptotic signals detected in the NS group using TUNEL assays aligned with our transcriptomics analysis (Figure 5). Overall, the differential autophagic and apoptotic responses to nitrite stress between the two families suggest that the degradation of misfolded proteins plays a crucial role in nitrite stress in *Penaeus vannamei.* The nitrite-resistant families seem to evade ER stress by promoting the degradation of misfolded proteins and supporting the processing of properly folded proteins to maintain the normal functioning of the biological system. In contrast, nitrite-susceptible families face challenges in effectively handling misfolded proteins, resulting in increased cytotoxicity and oxidative stress. Based on these findings, genomic technologies can be used in identifying gene markers linked to nitrite tolerance to speed up genetic enhancement of tolerance. Moreover, it is suggested to mate highly tolerant families with those displaying favorable traits to produce shrimp varieties that have both nitrite tolerance and high yield. These selective breeding efforts can greatly enhance the nitrite tolerance of shrimp.

Currently, this study has several limitations. First, the study is limited by its short 72 h duration. Therefore, the long-term effects of nitrite stress on sensitive and tolerant families remain uncertain. Previous reports indicate that both short-term and long-term nitrite stress induce oxidative stress responses, disrupt energy metabolism, and cause tissue damage in shrimp [10,25,28,48,80]. Short-term nitrite stress in shrimp triggers acute physiological responses, from which they can quickly recover [52]. However, prolonged exposure causes severe damage and metabolic disorders, impairing recovery and affecting growth and survival [81]. Furthermore, numerous differentially expressed genes (DEGs) were found to be enriched in protein processing within the endoplasmic reticulum, yet the specific mechanisms through which these genes influence autophagy- and apoptosis-related pathways remain unclear. Hence, more evidence is essential to confirm that autophagy and apoptosis helps in decreasing the stress of nitrite nitrogen in tolerant shrimp.

## 4. Material and Methods

### 4.1. Ethics Statement

All procedures for animal breeding, handling, and sampling in this study received approval from the Animal Care and Ethics Committee of Guangxi Academy of Fishery Sciences (Animal ethics approval number: GAFS2023005), thereby affirming the trustworthiness and ethical conduct of our research.

### 4.2. Animals and Treatment

The *Penaeus vannamei* (8–12 g) used in the experiment was sourced from the Shrimp Breeding Center of Guangxi Institute of Fishery Sciences. Over the course of 10 generations, families of both tolerant and sensitive of *Penaeus vannamei* were constructed through artificial selection. Specifically, at least 20 full-sib families of *Penaeus vannamei* originating from different breeding lines were established and selected annually to assess their resistance to nitrite stress under consistent conditions since 2014. Among the originated families, those exhibiting the highest survival rate after 96 h of nitrite exposure were classified as nitrite-tolerant families, whereas those with the lowest survival rate were categorized as nitrite-sensitive families. Prior to the commencement of the experiment, the shrimp were acclimated for 3 days before the experiment began and were reared in a 400 L tank, adhering to standard aquaculture water parameters (salinity 25‰, pH7.9 ± 0.1, temperature of 27 ± 0.5 °C, and dissolved oxygen level of 6 ± 0.5 mg/L). In order to reduce the influence of environmental factors, environmental factors (such as temperature, pH, and dissolved oxygen) and nitrite concentrations were corrected every 6 h during experiments. During the experimental period, the shrimp were fed commercial pellet feed twice daily at 8:00 and 20:00 (nutritional composition: 38% crude protein, 4% crude fat, 8% crude fiber, 16% ash and 11% moisture, supplied by Fujian Tianma Technology Group Corporation, Fujian, China). To ensure optimal rearing conditions, all uneaten feed and feces were removed regularly throughout the experiment, and 90% of the seawater was renewed daily.

### 4.3. Acute Nitrite Exposure and Sample Collection

To assess how acute exposure to nitrite affects families that are either sensitive or tolerant to nitrite, we randomly assigned 300 shrimp from each group into two subgroups, with each subgroup containing 150 shrimp. For the nitrite-sensitive families, one subgroup was maintained in normal seawater, designated as the control subgroup (SC), while the other subgroup experienced nitrite stress, referred to as the stress subgroup (SN). In parallel, the nitrite-tolerant families were categorized into the control subgroup (TC) and the stress subgroup (TN). The SN and TN subgroups were exposed to a nitrite level of 600 mg/L, achieved by the introduction of NaNO_2_ solutions, whereas the SC and TC subgroups were sustained in diluted natural seawater free from nitrite. Water was replaced daily at a rate of 90% to maintain a stable nitrite concentration. After 72 h of nitrite exposure, hemolymph was collected from three randomly chosen shrimp through the pericardial sinus using a sterile needle and syringe, into an equal volume of pre-chilled anticoagulant buffer (sodium citrate 26 mmol/L, citric acid 30 mmol/L, glucose 100 mmol/L, NaCl 140 mmol/L). Subsequently, the hepatopancreas was carefully extracted from the shrimp using sterile tweezers.

### 4.4. Shrimp Survival Assay

In order to compare the survival rates concerning nitrite resistance between two families, we divided the shrimp from the nitrite-sensitive and nitrite-tolerant groups into four sets, each containing 60 shrimp, as outlined in Section 2.3. The survival rate was calculated by counting the dead shrimp twice a day over a period of three days.

### 4.5. Histological Analysis

The hepatopancreas of the shrimp was carefully removed and promptly preserved in a 4% paraformaldehyde solution for a duration of 24 h. Subsequently, the samples were dehydrated through 80% concentrations of ethanol for 45 min, 90% for another 45 min, 95% for 30 min, and finally 100% for 30 min, respectively, followed by embedding in paraffin. Subsequently, the hepatopancreas samples were transferred to xylene solution to eliminate the residual ethanol and complete the transparency treatment. Following that, the samples were embedded in paraffin and allowed to fully solidify. Following this, the specimens were sliced into ultrathin sections measuring 5 μm and subjected to staining with hematoxylin and eosin (H&E). Ultimately, these sections were examined using a bright-field microscope (ECLIPSE 90i; Nikon Corporation, Tokyo, Japan). Under the microscope, healthy hepatopancreatic tissue display a regular and orderly pattern with closely arranged cells and clear boundaries. Any divergence from the typical structure might be a symptom of a disease or injury. Normal hepatopancreatic nuclei are typically round or oval in shape, displaying fine chromatin and a uniform red or pink staining. If dark or light areas are present, they could suggest abnormal cytoplasmic changes like vacuole formation or cytolysis. The hepatic tubules of the hepatopancreas should be neatly arranged, of the same size, and have a star-shaped lumen. If the hepatic tubules are disordered, of different sizes, or show vacuoles, then they may indicate tissue damage.

### 4.6. Assay of Oxidative Damage and Antioxidant Enzymes

The hepatopancreas described in Section 2.5 was blended with deionized water at a temperature of 4 °C. Following this, the mixture underwent centrifugation at 3500 rpm at 4 °C for a duration of 10 min. Afterward, the resulting supernatants from both the hepatopancreas and plasma were utilized to evaluate indicators of antioxidant capacity and oxidative stress. This included measurements of superoxide dismutase (SOD), total antioxidant capacity (T-AOC), alkaline phosphatase (AKP), acid phosphatase (ACP), nitrite levels, and malondialdehyde (MDA), based on the guidelines provided by commercial kits obtained from the Nanjing Jiancheng Institute of Biological Engineering in Nanjing, China.

### 4.7. Total Hemocyte Count (THC) Analysis

THC analysis was conducted by blood cell counting plate, following the protocol from previous research [82]. Hemolymph samples from each shrimp were extracted with a 1:1 (*v*/*v*) anticoagulant and the hemocyte count was then measured using a blood counting chamber (Qiujing, Shanghai, China) under a microscope.

### 4.8. Phagocytic Activity

Hemocytes were obtained using the same methodology previously described [53]. Subsequently, the hemocytes were first combined with 4% paraformaldehyde fixative and allowed to incubate at room temperature for 2 h. Afterwards, 20 µL of the hemocyte suspension was uniformly applied onto a slide, and the smears were left to dry at room temperature for 30 min. After fixation with methanol, the samples were treated with 5% Giemsa stain for 20 min and then rinsed with distilled water to remove excess stain. The samples were air-dried and observed under a microscope. The phagocytic activity was determined using the formula: PA = [(phagocytic hemocytes)/(total hemocytes)] × 100%, and evaluations were randomly conducted on a sample of 200 hemocytes.

### 4.9. Measurement of Intracellular ROS Contents

According to the instructions, the measurement of intracellular reactive oxygen species (ROS) was performed using a commercial assay kit (DCFH-DA, Beyotime, China). Briefly, hepatopancreatic cells were sourced by mincing the hepatopancreas in 1 mL of 0.01 M PBS (pH 7.2) using a sterile pestle after a 72 h exposure to nitrite stress. The resulting homogenate underwent filtration through a 150-μm steel mesh and was then centrifuged at 4 °C (150× *g* for 10 min) to obtain cell pellets. Hemocytes and hepatopancreatic cells were subsequently washed three times each with anticoagulant and PBS buffer. The ROS production was measured by combining 100 μL of both hemocyte and hepatopancreatic cell suspensions with 100 μL of a 1 μmol/mL 2′,7′-dichlorofluorescein diacetate (DCFH-DA) solution, followed by a 20-min incubation of the mixtures at 25 °C in darkness. The fluorescence intensity was recorded at an excitation wavelength of 488 nm and an emission wavelength of 525 nm, using a TECAN Infinite 200 PRO microplate reader (Tecan Austria GmbH, Victoria, Austria). Additionally, 100 μL of the cell suspension was lysed using 2× lysis buffer (50 mM Tris-HCl [pH 7.4], 300 mM NaCl, 2 mM EDTA, 2% NP-40, and 10% glycerol). Protein concentrations were measured with the BCA Protein Assay Kit (GenStar, Beijing, China). The levels of ROS in the samples were assessed by determining the fluorescence intensity per milligram of protein. All experiments included three biological replicates and three technical replicates.

### 4.10. Transcriptome Sequencing and Data Analysis

Total RNA was extracted from hemocytes and the hepatopancreas using TRIzol (Takara, Japan). The preparation of sequencing libraries was performed in accordance with the instructions provided by the manufacturer, utilizing the TruSeq™ RNA Sample Prep Kit (Illumina, San Diego, CA, USA). Sequencing was carried out on the Illumina Novaseq 6000 platform, with assistance from Majorbio Bio-pharm Technology Co., Ltd. (Shanghai, China). In summary, mRNA containing poly-A tails was extracted from the total RNA samples using oligo (dT) beads. This isolated mRNA was subsequently converted into double-stranded cDNA using random primers for reverse transcription. Following this, short cDNA fragments were linked with sequencing adapters and underwent sequencing on the Illumina Novaseq 6000 platform. To obtain clean reads, the raw reads were filtered using the Trimmomatic software (version 0.36) [83] and aligned to the reference genome of *Penaeus vannamei* (ASM378908v1) at the National Center for Biotechnology Information (NCBI) by Hisat2 software (version 2.2.1) with default parameters. Complete and accurate transcripts are generated after completing clean reads splicing using Cufflinks software (version 2.2.1). The longest sequences derived from the new transcript are clustered into unigenes and annotated using databases such as Pfam, NR, NT, COG, GO, and KEGG to obtain comprehensive gene annotation information. Differential expression analysis was conducted using the DESeq2 R package (Version 1.24.0) [84]. After standardizing the original *p*-values through false discovery rate (FDR) adjustment, differentially expressed unigenes (DEGs) were identified based on the criteria of an adjusted *p*-value (adj-*p*) less than 0.05 and a fold change exceeding 1.5. The functional information of DEGs was obtained by performing GO function enrichment analysis and KEGG pathway enrichment analysis using ClusterProfiler software (version 4.0). To verify the transcriptome, real-time quantitative PCR was performed on a selection of representative DEGs (see Figure S1). Comprehensive experimental protocols related to the comparative transcriptomic analyses are available in the Supporting Information.

### 4.11. Total RNA Extraction and Quantitative Real-Time PCR

Total RNA was isolated from shrimp tissues using TRIzol (Takara, Kusatsu, Japan) and reverse-transcribed into cDNA with a PrimeScript™ RT Reagent Kit (Perfect Real Time) (Takara, Beijing, China). The RNA samples were analyzed via 1.0% agarose gel electrophoresis and quantified at 260 nm, with all OD260/OD280 ratios ranging from 2.0 to 2.2. RT-qPCR was conducted on a QuantStudio 3 (Applied Biosystems, Foster City, CA, USA) to assess mRNA expression levels. All experiments were performed in triplicate for each group to measure fluorescence signal strength and obtain cycle threshold (Ct) values. These values were subsequently converted to fold differences using the 2^−ΔΔCt^ method for relative quantification, with β-actin serving as the endogenous control for mRNA. All primer sequences were listed in Supplementary Table S1.

### 4.12. Apoptotic Cell Analysis

Apoptosis was determined using terminal deoxynucleotidyl transferase-mediated dUTP nick end labeling staining (TUNEL) [85]. TUNEL staining was performed to evaluate the quantity of apoptotic nuclei in hemocytes and hepatopancreas following exposure to nitrite stress. Hemocytes and hepatopancreas were washed separately using anticoagulant and PBS, and subsequently fixed in 4% paraformaldehyde. After fixation, the samples were embedded in paraffin, deparaffinized, and washed, followed by treatment with a proteinase K working solution for 22 min at 37 °C. A membrane-permeabilizing solution (0.1% Triton) was then applied to the tissues and incubated for 20 min. The samples were subsequently counterstained with a DAPI solution in the dark and incubated for 5 min at room temperature. Following this, the samples were washed by immersing the slides in PBS solution three times, with each immersion lasting for 5 min at room temperature. Excess liquid was gently removed, and 20% glycerol was added to the sample area to maintain moisture. The sections were stained and processed according to the protocols provided by the TUNEL Apoptosis Detection Kit (BIOSSCI), and images were captured using a fluorescent microscope. Image analysis was conducted using Image J software (version 1.54p) for quantitative assessment. A comprehensive quantitative analysis was conducted by integrating cell morphological parameters, including nuclear area, perimeter, and shape factor, with content optical density measures, such as average optical density and integral optical density. Blue nuclei labeled with DAPI represent total cell counts, while bright green nuclei indicate apoptotic cells. The number of TUNEL-positive cells was determined, and the apoptosis index was calculated using the formula: apoptosis index = (number of apoptotic cells/total number of nuclei) × 100%.

### 4.13. Transmission Electron Microscopy (TEM)

Hemolymph was obtained from shrimp using disposable syringes filled with an equivalent volume of chilled anticoagulant solution. After collection, the hemolymph was centrifuged at 900× *g* for 10 min at a temperature of 4 °C to isolate hemocytes. The hepatopancreas, along with the hemocytes were subsequently fixed in 2.5% glutaraldehyde buffered with 0.1 M phosphate for 24 h at 4 °C. An additional fixation step was performed using 1% osmium tetroxide. After being rinsed with PBS, the specimens were dehydrated through a series of increasing concentrations of ethanol (30%, 50%, 70%, and 90%), followed by a series of graded acetone (90% and 100%). The samples were then immersed in a combination of epoxy resin and acetone. After drying, an ultra-thin microtome was used to section the samples, which were subsequently stained with uranyl acetate and double stained with lead citrate. Finally, the sections were preserved with a water-soluble sealant for further analysis using a HITACHI HT7800 transmission electron microscope (Hitachi, Tokyo, Japan). For TEM analysis, the number of apoptotic body was counted in SN and TN groups. All experiments were conducted in triplicate.

### 4.14. Statistical Analysis

Statistical analyses were conducted using GraphPad Prism version 8.0.2 for Windows. The normality of the distributions was verified through the Shapiro–Wilk test, while Bartlett’s test was used to assess homogeneity of variance. Data were evaluated using one-way analysis of variance (ANOVA) and two-way ANOVA, followed by Tukey’s multiple comparisons test. Normality and variance homogeneity were checked in all cases. Results are presented as means ± SD, with the number of replicates (n) indicated in the relevant figure legends. Significant differences between groups were denoted with “*” for *p* < 0.05 and “**” for *p* < 0.01.

## 5. Conclusions

**Figure 7:**
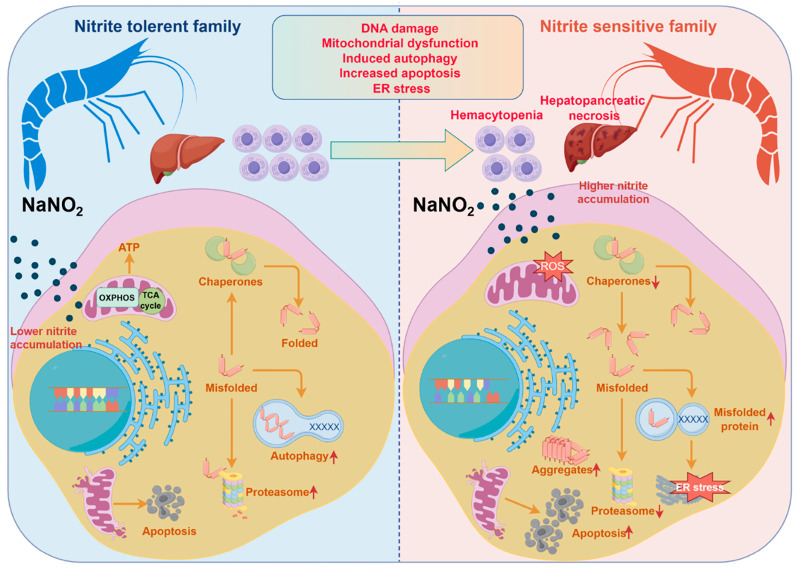
When subjected to nitrite stress, the nitrite-sensitive families demonstrated significantly greater physiological damage compared to their nitrite-tolerant counterparts. This damage was characterized by hepatic tubular deformation, hepatocyte necrosis, decreased hemocyte counts, reduced phagocytic activity of hemocytes, increased production of ROS and MDA, elevated nitrite accumulation, and inhibited activity of ACP and AKP enzymes. Furthermore, transcriptomic analysis revealed an upregulated activation of protein processing in the endoplasmic reticulum in the nitrite-tolerant families. It is hypothesized that nitrite-tolerant families can effectively eliminate misfolded proteins and induce autophagy to attenuate cell apoptosis and ER stress, thereby improving shrimp survival under nitrite stress. Up−regulated expression is represented by upward arrow, and down−regulated expression is represented by downward arrow. Figure was created with Figdraw (accessed on 16 October 2024, https://www.figdraw.com).

In summary, the current study showed that nitrite stress caused considerable hepatopancreatic damage and extensive mitochondrial structural changes in shrimp. Nonetheless, the nitrite-tolerant families can effectively eradicate misfolded proteins, trigger autophagy and apoptosis to attenuate cellular damage and oxidative stress, and improve survival of shrimp (Figure 7). These findings offer valuable insights into the mechanisms of nitrite stress tolerance in shrimp, with potential applications in advancing genetic breeding in shrimp aquaculture.

## Data Availability

The datasets supporting this article are included within the article and its Supplementary Information. Other details will be made available on reasonable request.

## Supplementary Materials

The following supporting information can be downloaded at: https://www.mdpi.com/article/10.3390/ijms26041641/s1.
