# Supplementary material for: Shrimp Shapes a Nitrite Tolerance Trait via Regulating Autophagy and Apoptosis
Source: Int J Mol Sci. 2025 Feb 14;26(4):1641. doi: 10.3390/ijms26041641 (PMC11855798; doi:10.3390/ijms26041641)
Supplement: Supplementary file 1 [file ijms-26-01641-s001.zip › ijms-3444979-supplementary/Supplementary Figs.pdf]

Supplementary Figures

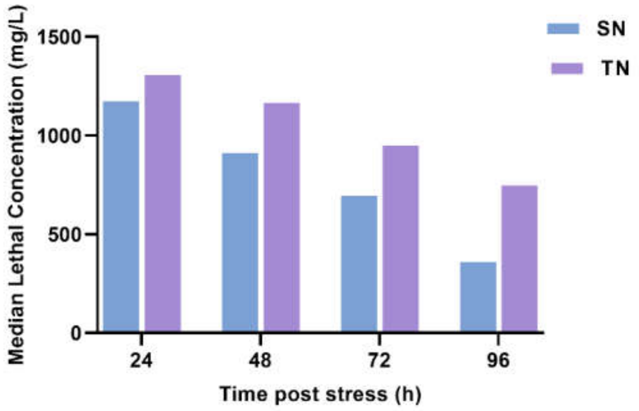

Fig. S1. Semi-lethal concentrations of nitrite sensitive and nitrite tolerant families at different time points under nitrite stress.

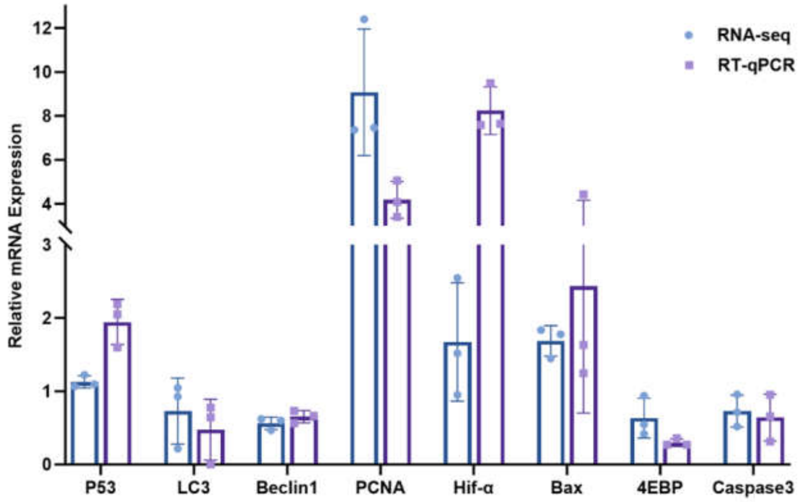

Fig. S2. Validation of expression profiles in transcriptome data by RT-qPCR for 8 DEGs from two populations.
